# Defining the nitrogen regulated transcriptome of *Mycobacterium smegmatis* using continuous culture

**DOI:** 10.1186/s12864-015-2051-x

**Published:** 2015-10-19

**Authors:** Michael Petridis, Andrej Benjak, Gregory M. Cook

**Affiliations:** Department of Microbiology and Immunology, Otago School of Medical Sciences, University of Otago, P.O. Box 56, Dunedin, New Zealand; Global Health Institute, École Polytechnique Fédérale de Lausanne, Lausanne, 1015 Switzerland; Maurice Wilkins Centre for Molecular Biodiscovery, The University of Auckland, Private Bag 92019, Auckland, 1042 New Zealand

**Keywords:** Mycobacteria, Nitrogen depletion, Continuous culture, RNA-sequencing, Gene expression profiling

## Abstract

**Background:**

Nitrogen is essential for microbial growth and its importance is demonstrated by the complex regulatory systems used to control the transport, assimilation and metabolism of nitrogen. Recent studies are beginning to shed light on how mycobacteria respond to nitrogen limitation and several regulators (e.g., GlnR, P_II_) have been characterized at a molecular level. However, despite this progress, our knowledge of the transcriptional response of mycobacteria to nitrogen limitation and its regulation is confined to batch culture.

**Methods:**

To gain further insight into the response of mycobacteria to nitrogen limitation, we developed a nitrogen-limited chemostat. We compared the transcriptional response of nitrogen-limited cells to carbon-limited cells using RNA-seq analysis in a continuous culture model at a constant growth rate.

**Conclusions:**

Our findings revealed significant changes in the expression of 357 genes (208 upregulated, 149 downregulated; >2-fold change, false discovery rate <5 %) in response to nitrogen limitation in continuous culture. The vast majority of the GlnR regulon (68 %) was differentially expressed under nitrogen limitation in continuous culture and approximately 52 % of the 357 genes overlapped with a previously published study investigating the response of *M. smegmatis* to nitrogen limitation in batch culture, while expression of only 17 % of the genes identified in batch culture were affected in our chemostat model. Moreover, we identified a unique set of 45 genes involved in the uptake and metabolism of nitrogen that were exclusive to our chemostat model. We observed strong downregulation of pathways for amino acid catabolism (i.e., alanine, aspartate, valine, proline and lysine), suggesting preservation of these amino acids for critical cellular function. We found 16 novel transcriptional regulators that were directly or indirectly involved in the global transcriptomic response of *M. smegmatis* to nitrogen limitation and identified several non-coding RNAs that might be involved in the transcriptional or post-transcriptional regulation of nitrogen-regulated gene expression.

**Results:**

Using nitrogen-limited continuous culture we identified the nitrogen-responsive transcriptome of M. smegmatis, including a number of small non-coding RNAs implicated in controlling nitrogen-regulated gene expression.

**Electronic supplementary material:**

The online version of this article (doi:10.1186/s12864-015-2051-x) contains supplementary material, which is available to authorized users.

## Background

Carbon and nitrogen are key components of organic material and their availability in the environment is necessary for growth and survival of microorganisms. The major mechanisms of carbon source utilization and its regulation are well defined for many groups of microorganisms, but our knowledge of nitrogen metabolism and its regulation is largely confined to enteric bacteria (for review see [1–3]). The importance of nitrogen for microbial growth is demonstrated by the complex regulatory systems used to control nitrogen assimilation in response to internal and external nitrogen levels. In some bacteria, nitrogen availability is sensed by the intracellular ratio of 2-oxoglutarate:glutamine [4]. This signal ensures that the uptake of nitrogen sources are commensurate with the metabolic requirements of the organism i.e. carbon or nitrogen. High 2-oxoglutarate levels signal nitrogen limitation, while high glutamine levels signal nitrogen excess in bacteria [1, 4, 5].

In enteric bacteria, nitrogen metabolism is regulated by the two-component regulatory system NtrBC and the signal transduction protein P_II_ [6]. These regulatory systems coordinate nitrogen metabolism by regulating genes involved in ammonium assimilation, amino acid transport and nitrate metabolism through protein-protein interactions [1, 4, 6, 7]. An integral component of this regulation is GlnD, an uridylyl transferase/uridylyl-removing enzyme that senses and transmits the nitrogen status of the cell to the P_II_ protein [8]. The P_II_ protein in its uridylylated state stimulates phosphorylation of the response regulator NtrC by the NtrB kinase, which results in expression of the NtrBC-regulated genes under nitrogen limitation [9]. Homologues of the P_II_ protein are found in many bacterial genomes, where they are mainly involved in the regulation of nitrogen metabolism. However, the P_II_ protein may have additional roles, as recently described in cyanobacteria, where it regulates the metabolism of inorganic carbon [10].

Actinomycetes are a group of Gram-positive bacteria that inhabit a wide range of aquatic, terrestrial and human habitats. In actinobacteria, two different regulatory systems have been identified that regulate the transcriptional response to nitrogen limitation, AmtR, a TetR-type transcriptional regulator and GlnR, an OmpR-type transcriptional regulator [11–13]. AmtR was identified in *Corynebacterium glutamicum* to regulate more than 30 genes involved in nitrogen metabolism, including the P_II_ protein, glutamine synthetase, glutamate synthase and urease [12]. In *Streptomyces coelicolor*, GlnR mediates the transcriptional response to nitrogen limitation by elevating expression of the *amtB*-*glnK*-*glnD* operon and the glutamine synthetase *glnA*, while repressing transcription of the glutamate dehydrogenase *gdhA* [13, 14].

Slow-growing mycobacteria such as *Mycobacterium tuberculosis* appear to harbor only GlnR that was shown to regulate the expression of at least 33 genes in response to nitrogen limitation [15]. *M. tuberculosis* can exploit different nitrogen sources, however, organic nitrogen sources (e.g. asparagine and aspartate) are preferred for extracellular and intracellular growth [16, 17]. Amino acids can function as both carbon and nitrogen sources, and although *M. tuberculosis* can metabolize a variety of amino acids *in vitro*, only a small number (aspartate, glutamate, asparagine and glutamine) can support growth of *M. tuberculosis* at the acidic pH that prevails in host macrophages [18]. Inorganic nitrogen sources such as ammonium and nitrate are less efficient for growth than amino acids [18], but the reasons for this remain unknown.

The genome of the fast growing saprophytic actinobacterium *Mycobacterium smegmatis* contains copies of both global nitrogen regulators, GlnR and AmtR [19]. It has recently been demonstrated that GlnR regulates the expression of more than one hundred genes in response to nitrogen limitation in *M. smegmatis* [20], while the AmtR regulon remains unknown. Previous microarray studies using batch culture nitrogen run out experiments have identified 1090 genes in *M. smegmatis* that are differentially expressed in response to nitrogen limitation [21]. However, only a small subset of these genes is under control of GlnR, while the regulatory mechanisms for the majority have yet to be identified [20, 21]. During nitrogen run out studies, the growth rate changes significantly as nitrogen becomes depleted and therefore these types of experiments may not unequivocally uncover genes that respond solely to nitrogen limitation.

The aim of the current study was to deliver a molecular framework for how a mycobacterial cell responds to nitrogen-depleted and -replete conditions at a defined growth rate. To address this aim, we developed a nitrogen-limited chemostat for *M. smegmatis* at a constant growth rate of 0.12 h^−1^ (t_d_ = 5.7 h) followed by RNA-seq analysis to identify genes responding to nitrogen limitation.

## Results and discussion

### Development and validation of nitrogen-limited continuous culture to understand the global transcriptomic response to nitrogen limitation

The first step of this study was to establish a defined minimal medium limited for either nitrogen or carbon to identify the molecular response of *M. smegmatis* to nitrogen limitation. We modified HdB medium [22] by replacing 0.05 % (w/v) Tween-80 with 0.05 % (w/v) Tyloxapol, which cannot be metabolized as a carbon source, and (NH_4_)_2_SO_4_ with NH_4_Cl and K_2_SO_4_ (11.4 mM) to avoid simultaneous limitation for sulphur and nitrogen. Inorganic nitrogen sources (e.g. ammonium) are less efficient for growth than amino acids, however, we chose an inorganic nitrogen source to allow tighter control of the nitrogen to carbon ratio in the medium. Using batch culture, we identified glycerol as a suitable carbon source, with a high cell yield and no change in external pH during the course of the experiment compared to other carbon sources such as glucose, acetate or succinate (Additional file 1, Figure S1A). When cells were grown in batch culture on glycerol (25 mM) under nitrogen-replete conditions, growth ceased at an optical density (OD_600_) of 3.5 (Additional file 1, Figure S1B). When the culture was limited for nitrogen (carbon excess), the final OD_600_ was 2.1 (Additional file 1, Figure S1B). Based on these experiments we used 30 mM glycerol and 10 mM NH_4_Cl (nitrogen-replete)/1.25 mM NH_4_Cl (nitrogen-depleted) for continuous culture studies.

**Fig. 1:**
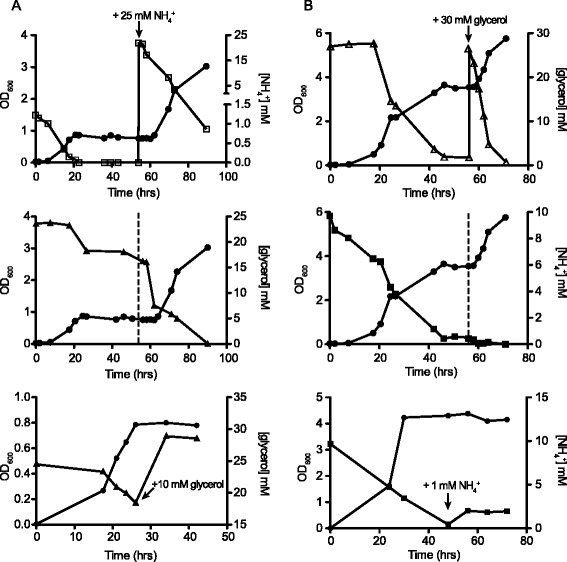
Growth of *M. smegmatis* in continuous culture under nitrogen-depleted and nitrogen-replete conditions. Optical density (OD_600_), residual glycerol and ammonium concentrations in the medium were monitored during the experiment. **a** The effect of a pulse with 25 mM NH_4_Cl on residual ammonium (open squares) and glycerol (closed triangles) concentrations in the medium and OD_600_ (closed circles) in a nitrogen-depleted culture. A pulse with 10 mM glycerol had no effect in a nitrogen-depleted culture. **b** The effect of a pulse with 30 mM glycerol on residual glycerol (open triangles) and ammonium (closed squares) concentrations in the medium and OD_600_ (closed circles) in a nitrogen-replete culture. A pulse with 1 mM NH_4_Cl had no effect on the nitrogen-replete culture, respectively

In continuous culture, *M. smegmatis* was grown at 50 % air saturation and at a dilution rate of 0.12 h^−1^ until steady-state conditions were achieved (typically after 3–4 resident times). Nitrogen-limited cultures (1.25 mM NH_4_Cl/30 mM glycerol) reached steady-state at an OD_600_ of 0.8 and no residual ammonium could be detected (Fig. 1a, top). The residual concentration of glycerol in the medium was approximately 18 mM indicating cells were not carbon limited (Fig. 1a, middle). At this stage, addition of 25 mM NH_4_Cl into the culture vessel led to an immediate increase in optical density, demonstrating that the cultures were indeed limited for nitrogen (Fig. 1a, top). Addition of 10 mM glycerol to a nitrogen-depleted culture had no effect (Fig. 1a, bottom). In the carbon-limited culture (10 mM NH_4_Cl/30 mM glycerol), the steady-state-phase was reached after 56 h at an OD_600_ of 4.3 with the residual ammonium concentration in the medium remaining constant at 0.5-1 mM (Fig. 1b, middle). A pulse of 1 mM NH_4_Cl into the culture vessel had no effect on growth (Fig. 1b, bottom), while the addition of glycerol led to a significant increase in OD_600_, demonstrating nitrogen excess (replete) under these conditions (Fig. 1b, top).

For each condition, three independent biological replicates were grown until steady-state-phase and cells were harvested after at least 3 volume changes in the chemostat vessel. The analysis of the transcriptome using RNA-sequencing revealed a total of 357 differentially expressed genes under nitrogen limitation [>2-fold change, false discovery rate (FDR) was <5 %] i.e. approximately 5 % of the *M. smegmatis* genome (Additional file 2, Table S1). Moreover, the usage of different thresholds (i.e. 2-fold, <1 % FDR; 2-fold, <0.1 % FDR; 1.5-fold, <0.1 % FDR) had no major effect on the number of differentially expressed genes. In total, 208 genes were upregulated and 149 were downregulated. Classification of these genes into functional categories showed major changes in transport proteins, genes that are associated with nitrogen and amino acid metabolism and genes that are functionally assigned as regulatory proteins. A large number of genes encoding for hypothetical proteins were differentially expressed (36 upregulated; 38 downregulated), however, their function in response to nitrogen depletion needs to be investigated (Additional file 3, Figure S2).

### Nitrogen limitation studies in continuous culture versus batch culture

**Fig. 2:**
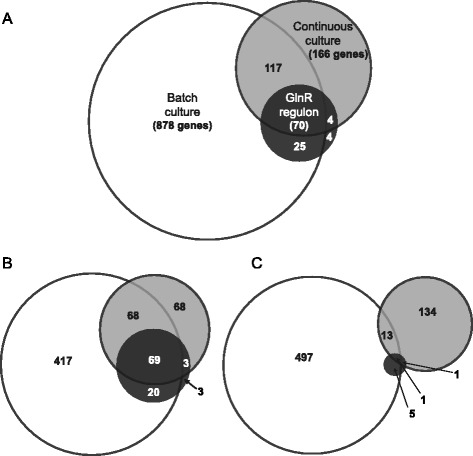
Distribution of differentially expressed genes comparing nitrogen-depleted continuous culture versus nitrogen-depleted batch culture [21]. In this comparison we included genes that were reported to be under control of GlnR [20]. **a** Included are all genes that were reported to be differentially expressed in continuous culture and batch culture. **b** Upregulated genes that are upregulated in continuous culture and batch culture and activated by GlnR. **c** Downregulated genes that are downregulated in continuous culture and batch culture and are repressed by GlnR. Numbers indicate the total number of genes that fall into the respective category

Previous work published by Williams et al*.* focused on the response of *M. smegmatis* to nitrogen stress in batch culture nitrogen run out experiments and showed differential expression of 1090 genes (574 upregulated, 516 downregulated) (Fig. 2) [21]. Surprisingly, 903 of these genes were not differentially expressed in our continuous culture. In fact, only 17 % of the genes reported by Williams et al*.* responded to nitrogen limitation in continuous culture, including 70 genes that were predicted to be under control of GlnR (Fig. 2a) [20]. Despite the differences in methodology between the two studies, we were able to identify a significant overlap in nitrogen metabolism related genes. For example, expression of a similar set of ammonium and nucleotide uptake systems (Table 1) and metabolic pathways (Table 2) were elevated in both studies. In addition, we identified a unique set of genes involved in the metabolism of amino acids, nucleotides and urea that were nitrogen-responsive in continuous culture (Table 2). We identified 36 additional genes, including those encoding for regulatory enzymes, which showed an inversed expression profile in continuous culture compared to batch culture (Additional file 4, Table S2). In batch culture it is often difficult to assign transcriptional changes to a single stimulus due to changes in growth rate, nutrient depletion and end-product buildup. It is striking that there was very little overlap in the downregulated genes in response to nitrogen limitation between batch culture and continuous culture (Fig. 2c). A major portion of the 497 downregulated genes in batch culture was associated with the general reduction in cellular metabolism due to a reduced growth rate. Development of a nitrogen-limited continuous culture enabled us to define transcriptional changes purely in response to nitrogen limitation excluding growth rate effects. We showed that using continuous culture we can identify the set of genes involved in nitrogen uptake and metabolism that were reported by Williams et al., however, this system allowed us to further extend our knowledge towards the transcriptional response to nitrogen limitation and reduce the number of genes responding to other environmental factors [21].

**Table 1:** Differentially expressed genes that are involved in uptake of nitrogen compounds during nitrogen limitation

Predicted transported substrate	mc^2^155 locus^a^	Expression ratio^b^	FDR^c^	Description
Amino acids	msmeg_2525	13.18	8.41E-26	amino acid permease
	msmeg_2184	7.09	4.86E-11	amino acid permease
	msmeg_6735	6.11	1.56E-13	amino acid permease
	msmeg_2981	5.81	5.53E-29	branched-chain amino acid ABC transporter permease
	msmeg_1613^d^	4.88	3.38E-07	polar amino acid ABC transporter inner membrane protein
	msmeg_3231	2.16	1.80E-02	cysteine ABC transporter permease/ATP-binding protein
	msmeg_6876	2.14	9.32E-03	branched-chain amino acid transport ATP-binding protein
	msmeg_3203^d^	0.33	4.71E-05	transporter LysE family protein
Ammonium	msmeg_4635	7.49	9.82E-37	ammonium transporter
	msmeg_6259	2.70	2.06E-04	ammonium transporter
	msmeg_2425	2.70	3.95E-11	ammonium transporter
Nucleotides	msmeg_2570	6.79	4.33E-08	xanthine / uracil permease
	msmeg_5730	6.55	1.50E-26	permease for cytosine / purines / uracil / thiamine / allantoin
	msmeg_4011	6.30	2.56E-12	pyrimidine permease RutG
	msmeg_6660	4.22	1.88E-17	permease for cytosine / purines / uracil / thiamine / allantoin
	msmeg_1177	4.22	4.90E-08	cytosine / purines / uracil / thiamine / allantoin permease
	msmeg_1293	3.98	1.09E-17	xanthine / uracil permease
	msmeg_3402	2.23	3.57E-06	cytosine permease
Nitrate	msmeg_0433	3.67	1.73E-04	nitrite extrusion protein

^a^Locus number of gene in *M. smegmatis* mc^2^155

^b^Mean gene expression ratio of three biological replicates

^c^ *P*-values of gene expression ratio from three biological replicates were corrected for multiple testing using the Benjamini and Hochberg False Discovery Rate (FDR)

^d^Genes in the nitrogen regulated transcriptome that were discovered in this study

**Table 2:** Genes involved in nitrogen metabolism that are differentially expressed during nitrogen limitation in *M. smegmatis* mc^2^155

Metabolic pathway	mc^2^155 locus^a^	Expression ratio^b^	FDR^c^	Description
Amino acids	msmeg_2526	8.68	7.15E-35	tyramine oxidase
	msmeg_3993	5.19	3.03E-14	Asp/Glu racemase
	msmeg_0567	4.89	2.27E-12	selenophosphate synthetase
	msmeg_3973	3.85	3.71E-05	N-methylhydantoinase
	msmeg_4459^d^	3.22	1.59E-03	agmatinase
	msmeg_3317	3.06	1.59E-08	dihydrodipicolinate reductase N-terminal domain-containing protein
	msmeg_6261	2.73	4.86E-04	glutamine amidotransferase
	msmeg_6260	2.63	1.54E-04	glutamine synthetase
	msmeg_2493	2.42	3.78E-03	aminotransferase class I and class II family protein
	msmeg_6197^d^	2.40	1.58E-02	diaminopimelate decarboxylase
	msmeg_2494	2.20	8.20E-03	Xaa-Pro aminopeptidase
	msmeg_5374	2.18	4.69E-05	glutamate-ammonia ligase
	msmeg_6263	2.07	1.21E-02	glutamate synthase
	msmeg_2100^d^	2.02	9.01E-03	peptidase M20/M25/M40
	msmeg_6256^d^	0.49	7.47E-06	aspartate-semialdehyde dehydrogenase
	msmeg_5612^d^	0.40	1.25E-07	amino acid acetyltransferase
	msmeg_2691^d^	0.40	6.92E-03	N-acetylglutamate synthase
	msmeg_1762^d^	0.32	4.60E-07	piperideine-6-carboxylic acid dehydrogenase
	msmeg_5454^d^	0.28	7.84E-08	choloylglycine hydrolase
	msmeg_5119^d^	0.25	5.76E-09	1-pyrroline-5-carboxylate dehydrogenase
	msmeg_0019^d^	0.21	5.06E-23	amino acid adenylation protein
	msmeg_5117^d^	0.19	5.23E-07	proline dehydrogenase
	msmeg_1414^d^	0.19	5.97E-03	amidinotransferase
	msmeg_1413^d^	0.18	1.92E-05	ornithine-oxo-acid transaminase
	msmeg_0022^d^	0.18	3.49E-28	L-ornithine 5-monooxygenase
	msmeg_0021^d^	0.18	1.83E-21	aspartate alpha-decarboxylase
	msmeg_1764^d^	0.13	1.45E-03	L-lysine aminotransferase
Nucleotides	msmeg_4012	9.42	1.31E-24	phenylhydantoinase
	msmeg_5729	7.24	5.08E-19	hydantoin racemase
	msmeg_1294	3.89	2.39E-17	OHCU decarboxylase
	msmeg_1296	3.78	2.28E-07	uricase
	msmeg_2748	3.69	1.44E-05	soluble pyridine nucleotide transhydrogenase
	msmeg_1298	3.67	6.88E-12	guanine deaminase
	msmeg_3996	3.53	3.56E-13	phenylhydantoinase
	msmeg_1295	3.52	2.55E-12	transthyretin
	msmeg_3553^d^	2.95	1.98E-04	phenylhydantoinase
	msmeg_6116	2.64	1.09E-08	OHCU decarboxylase
	msmeg_3473	2.55	6.53E-03	uracil phosphoribosyltransferase
	msmeg_5727	2.21	4.92E-06	allantoicase
Urea	msmeg_2187	3.33	5.04E-06	urea amidolyase
	msmeg_1425^d^	2.60	2.80E-08	creatininase subfamily protein
	msmeg_3623	2.45	1.22E-06	urease accesory protein UreG
	msmeg_0435^d^	2.40	6.48E-06	allophanate hydrolase subunit 2
	msmeg_0436^d^	2.17	1.19E-04	allophanate hydrolase subunit 1
	msmeg_2189	2.16	9.92E-03	allophanate hydrolase
	msmeg_3624	2.11	1.40E-03	urease accesory protein UreF
	msmeg_3627	2.06	3.27E-04	urease subunit γ
C-N bond	msmeg_5358	7.23	9.62E-35	acetamidase/formamidase
	msmeg_5359	4.64	6.37E-14	cyanate hydratase
	msmeg_0571	4.06	1.08E-17	carbon-nitrogen hydrolase
	msmeg_3995	3.99	1.09E-13	N-carbamoyl-L-amino acid amidohydrolase
	msmeg_6266	3.68	5.45E-18	thiocyanate hydrolase subunit β
	msmeg_4367	3.67	2.87E-17	formamidase
	msmeg_6267	3.42	2.79E-15	thiocyanate hydrolase subunit γ
	msmeg_0566	2.96	3.15E-06	aliphatic amidase
	msmeg_4381	2.58	1.56E-08	amidase
	msmeg_3403	2.38	2.83E-06	formamidase
	msmeg_5373	2.35	1.43E-04	nitrilase 2
	msmeg_0443^d^	2.28	1.82E-02	carbon-nitrogen hydrolase
	msmeg_3397	2.26	3.75E-04	acetylornithine deacetylase
	msmeg_2491	2.25	3.45E-02	acetylornithine deacetylase
Nitrate/Nitrite	msmeg_0427	4.42	1.52E-03	nitrite reductase, large subunit
	msmeg_0332^d^	0.16	7.18E-10	2-nitropropane dioxygenase
CoA biosynthesis	msmeg_6097^d^	0.49	9.14E-05	pantoate-β-alanine ligase

^a^Locus number of gene in *M. smegmatis* mc^2^155

^b^Mean gene expression ratio of three biological replicates

^c^ *P*-values of gene expression ratio from three biological replicates were corrected for multiple testing using the Benjamini and Hochberg False Discovery Rate (FDR)

^d^Genes in the nitrogen regulated transcriptome that were discovered in this study

### Nitrogen limitation activates the expression of genes involved in scavenging nitrogen sources in the environment

In this study, a total of 53 genes encoding for transporters or corresponding binding proteins were differentially expressed in response to nitrogen limitation (Additional file 2, Table S1). Of the upregulated genes, 18 encoded for transporters that are generally involved in uptake of both organic and inorganic nitrogen-containing compounds like amino acids (e.g. *msmeg_2525*), nucleotides (e.g. *msmeg_5730*) and ammonium (e.g. *msmeg_4635*) (Table 1). Previously published data by Berney and Cook showed downregulation of nine of these transporters in a carbon-limited continuous culture comparing slow growth (t_d_ 69 h) versus fast growth (t_d_ 4.6 h) [23]. All three ammonium transporters and several amino acid (3 of 8) and nucleotide transporters (3 of 7) showed a significant decrease in transcript level, however transcription of all of these genes was enhanced upon nitrogen limitation. The GlnR regulon comprises 15 of these nitrogen compound transporters including the three ammonium transporters and the majority of nucleotide and amino acid transporters. The most prominent amino acid permease (*msmeg_2525*) was not under direct control of GlnR. In continuous culture, we identified 4.8-fold upregulation of a polar amino acid ABC transport system that has not been reported to be affected by nitrogen limitation. Further work is required, to identify the regulatory mechanisms that mediate the uptake of amino acids under nitrogen depletion. We observed a decrease in gene expression for the catabolism of five amino acids (Table 2), suggesting *M. smegmatis* attempts to balance the availability of ammonium and particular amino acids under nitrogen depletion. This finding is supported by a recent report of a proteasome-mediated amino acid recycling in *M. smegmatis* under nutrient limitation [24]. The transcriptomic response of *M. smegmatis* to increase its ability for the uptake (scavenging) of nitrogen compounds is remarkably different from other actinobacteria like *C. glutamicum*, where no amino acid permeases were upregulated under nitrogen limitation [25].

**Fig. 3:**
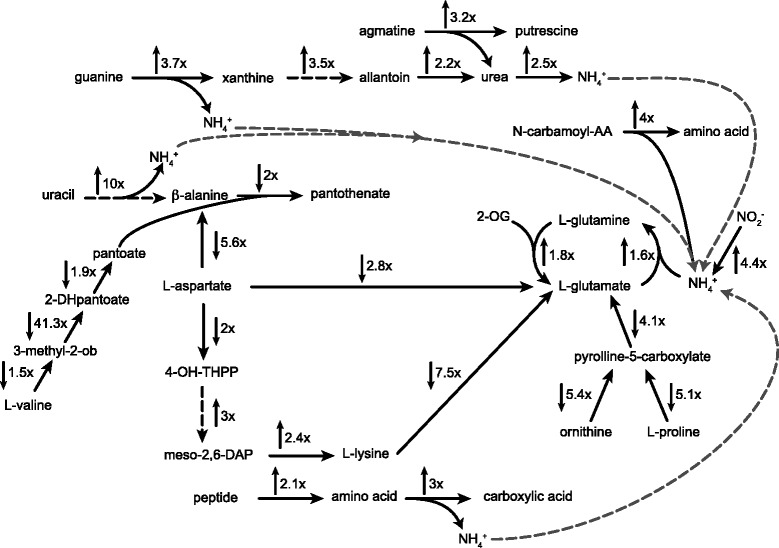
Diagram of pathways involved in nitrogen metabolism during nitrogen limitation in *M. smegmatis*. Shown are selected metabolic pathways that are discussed. Fold change in gene expression and predicted directionality of reaction are indicated by numbers and arrows. Solid black arrows indicate one-step reactions and dotted black arrows indicate multi-step reactions. 2-DHpantoate: 2-dehydropantoate; 2-OG: 2-oxoglutarate; 3-methyl-ob: 3-methyl-2-oxobutanoate; 4-OH-THPP: 4-hydroxy-2,3,4,5-tetrahydrodipicolinate; AA: amino acid; meso-2,6-DAP: meso-2,6-diaminopimelate; NH_4_^+^: ammonium; NO_2_^−^: nitrite

Peptides appear to play an important role in replenishing the intracellular ammonium pool necessary for the different anabolic pathways in *M. smegmatis* (Table 2), (Fig. 3). Amongst the genes induced in response to nitrogen limitation we identified a peptidase, an aminopeptidase and an aliphatic amidase catalyzing the successive degradation of peptides to amino acids and the subsequent recovery of ammonium (Table 2). Furthermore, expression of a gene encoding the dipeptide transporter DppB (*msmeg_1085*) was induced 7.7-fold (FDR < 0.1 %). The dipeptide transporter facilitates the import of dipeptides into the cytoplasm, where they can be hydrolyzed in order to replenish the intracellular amino acid pool or serve for incorporation into proteins [26]. The importance of (di-)peptides as nitrogen donors in mycobacteria remain to be explored, whereas previous work suggested that peptides cannot be used as carbon source [27].

### Strong induction of nucleotide catabolism and recovery of ammonium

In our analysis we identified a novel set of genes that were significantly upregulated upon nitrogen limitation and appeared to be involved in the breakdown of nucleotides and inorganic nitrogen sources (Table 2). Nucleotides contain a high level of nitrogen and their degradation plays an important role in nitrogen metabolism in a large number of different microorganisms (*e.g. Bacillus subtilis*), where purine catabolic pathways are described as an alternative pathway of nitrogen utilization once primary nitrogen sources are exhausted [28]. Genes encoding for proteins involved in the degradation of purine nucleotides were previously reported to be upregulated in *M. smegmatis* during nitrogen limitation [21]. The enzyme guanine deaminase is upregulated 3.7-fold (FDR < 0.1 %) catalyzing the reaction from guanine to xanthine and releasing 1 mol of ammonium per 1 mol guanine. The three enzymes, uricase (up 2.8-fold, FDR < 0.1 %), transthyretin (up 3.5-fold, FDR < 0.1 %) and 2-oxo-4-hydroxy-4-carboxy-5-ureidoimidazoline decarboxylase (up 3.9-fold, FDR < 0.1 %) catalyze the subsequent steps of urate degradation resulting in the production of allantoin. This metabolite is then further degraded with the concomitant production of ureidoglycolate and urea, which is broken down in either a one-step reaction via urease or a two-step reaction via urea carboxylase/allophanate hydrolase enzyme complex to ammonium (Fig. 3). Transcription of the urease-encoding genes was elevated also in the batch culture, while genes encoding for an allophanate hydrolase (*msmeg_0435*-*msmeg_0436*) were upregulated only under nitrogen depletion in continuous culture [21].

We observed an upregulation of the pyrimidine nucleotide degradation gene cluster and detected differential expression of a third copy of a putative phenylhydantoinase (*msmeg*_*3553*; up 3-fold, FDR < 0.1 %) that plays an important role in the degradation of pyrimidine nucleotides. Catabolism of pyrimidine nucleotides can occur through three different pathways; a reductive pathway in which uracil is reduced to two molecules of CO_2_ and one molecule of ammonium and β-alanine, respectively, an oxidative pathway and a recently identified Rut pathway [29]. *M. smegmatis* harbors a reductive uracil degradation pathway [30], which is induced upon nitrogen limitation. However, not all of the genes in this pathway have been identified in *M. smegmatis*. After the import of uracil by several nucleotide permeases, the first step of this reductive pathway is catalyzed by a dihydropyrimidine dehydrogenase that is able to reduce uracil and thymine to 5,6-dihydrouracil and 5,6-dihydrothymine (Fig. 3), followed by a reaction catalyzed by a phenylhydantoinase. This enzyme hydrolyzes the opening of the heterocyclic ring and β-alanine synthase (*msmeg_3555*; up 1.9-fold, FDR < 1 %) is catalyzing the last step of this pathway (Fig. 3).

### Amino acid catabolism is strongly downregulated under nitrogen limitation

A large cohort of genes, particularly in the catabolism of the amino acids alanine, aspartate, valine, proline and lysine were downregulated 5-40-fold in response to nitrogen depletion (Table 2), (Fig. 3). Interestingly, most of these genes showed an elevated transcription in batch culture (Additional file 5, Table S3). The amino acid alanine has a substantial importance as a central metabolite in bacterial metabolism and also its role in the synthesis of peptidoglycan as D-/L-alanine is essential for bacteria. Several pathways have been described contributing to the biosynthesis of L-alanine and β-alanine, e.g. catabolism of amino acids (i.e. valine and cysteine) and transamination of pyruvate. In *M. smegmatis*, genes encoding for all of the aforementioned pathways were differentially expressed in response to nitrogen limitation (Table 2). The conformation of the amino acid β-alanine does not allow its incorporation into proteins, but it serves together with pantoate as precursor of coenzyme A (CoA) biosynthesis, which is essential for a functional TCA cycle, as well as fatty acid and cholesterol biosynthesis. Degradation of purine nucleotides via an L-aspartate-alpha-decarboxylase (PanD) results in production of β-alanine and PanD was identified as the predominant pathway of β-alanine synthesis in the closely related *C. glutamicum*, where a *panD* mutant exhibited β-alanine auxotrophy [31]. However, expression of *panD* was strongly downregulated in *M. smegmatis* under nitrogen limitation. Valine degradation via the intermediates 3-methyl-2-oxobutanoate and 2-dehydropantoate to (R)-pantoate was repressed the same time, suggesting the demand to prevent unnecessary consumption of amino acids for CoA biosynthesis (Fig. 3).

A second pathway of L-aspartate (Asp) catabolism was differentially expressed in *M. smegmatis* under nitrogen limitation, which is linked to the concomitant biosynthesis of lysine. This pathway is a nine-step reaction including important metabolites such as L-aspartate semialdehyde (homoserine biosynthesis) and meso-2,6-diaminopimelate (constituent of bacterial cell walls). Interestingly, the initial steps of aspartate catabolism were repressed, while the degradation of meso-2,6-diaminopimelate to lysine was upregulated (Fig. 3). Lysine can act as donor of an amino group by transferring an ammonium group to 2-oxoglutarate to form glutamate under nitrogen excess, however, this pathway of lysine catabolism via a lysine aminotransferase is downregulated 7.5-fold (FDR < 1 %), indicating an intracellular accumulation of aspartate and lysine and suggesting a secondary function of these amino acids [32, 33]. Previous studies discussed the importance of intracellular lysine to control growth rate in mycobacteria and suggested a link between lysine accumulation and fatty acid metabolism [34]. Proline has been shown to serve as mechanism for methylglyoxal detoxification, when anabolic and catabolic processes were imbalanced and we show that proline degradation was repressed under nitrogen depletion.

### A large number of transcriptional regulatory systems are differentially expressed in response to nitrogen limitation

We identified 26 differentially expressed transcriptional regulators that are either directly or indirectly responding to nitrogen limitation in *M. smegmatis* (Table 3). Only a handful of these regulators have been characterized, including the nitrogen regulatory protein P_II_ (*msmeg_2426*) and the P_II_ adenylyl transferase (*msmeg_2427*). The mycobacterial copy of the P_II_ protein is not required for the regulation of the glutamine synthetase activity and does not act as regulator of the transcriptional response to nitrogen limitation [35]. This is in contrast to *C. glutamicum*, where the P_II_ protein was identified as the sole signal transduction protein, binding to AmtR and releasing this repressor from its target DNA, in order to allow transcription of genes involved in nitrogen uptake, assimilation and metabolism [12].

**Table 3:** Genes involved in regulatory mechanisms in response to nitrogen limitation in *M. smegmatis* mc^2^155

mc^2^155 locus^a^	Expression ratio^b^	FDR^c^	Description
***msmeg_1082***	7.04	1.06E-08	two-component regulator HTH luxR-type DNA binding domain
**msmeg_3997**	3.46	4.55E-15	regulatory protein
*msmeg_5731*	3.44	1.87E-07	GntR family transcriptional regulator
***msmeg_1597***	3.29	1.59E-14	transcription factor WhiB
msmeg_6236	3.27	1.32E-12	two-component system - regulatory protein
msmeg_6238	2.80	2.81E-10	two-component system - sensor kinase
***msmeg_2427***	3.19	2.01E-14	P_II_ uridylyl-transferase
***msmeg_2426***	2.18	5.48E-07	nitrogen regulatory protein P_II_
*msmeg_4006*	3.11	1.56E-08	CdaR family transcriptional regulator
msmeg_4368	3.11	1.49E-13	regulatory protein - FmdB family
msmeg_3297	2.78	1.21E-05	CadC family transcriptional regulator
msmeg_3298	2.36	4.75E-03	response regulator receiver domain-containing protein
msmeg_6198	2.41	1.85E-05	DNA-binding protein
msmeg_6824	2.16	1.03E-02	MarR family transcriptional regulator
**msmeg_0778**	2.11	1.33E-02	transcriptional regulator
msmeg_1420	2.07	1.46E-02	transcriptional regulatory protein
msmeg_5673	0.49	9.39E-05	transcriptional regulator
msmeg_3177	0.49	4.19E-02	transcriptional regulatory protein
msmeg_4394	0.48	5.57E-04	LysR family transcriptional regulator
msmeg_6789	0.45	2.14E-02	GntR family transcriptional regulator
msmeg_0473	0.38	2.21E-04	LuxR family transcriptional regulator
msmeg_5987	0.35	9.99E-03	two-component regulator
msmeg_6555	0.33	6.11E-07	TetR family transcriptional regulator
msmeg_0051	0.31	1.59E-05	transcription factor WhiB family protein
msmeg_1953	0.27	1.77E-06	transcription factor WhiB
msmeg_0622	0.19	4.57E-08	DNA-binding protein

Bold: within GlnR regulon; *Italic*: differentially expressed in the same direction in batch culture versus continuous culture; Underlined: inversed expression in batch culture versus continuous culture

^a^Locus number of gene in *M. smegmatis* mc^2^155

^b^Mean gene expression ratio of three biological replicates

^c^ *P*-values of gene expression ratio from three biological replicates were corrected for multiple testing using the Benjamini and Hochberg False Discovery Rate (FDR)

Another well-described transcriptional regulator is the OmpR-type response regulator GlnR, which has been identified as a mediator of the transcriptomic response to nitrogen limitation in *M. smegmatis* [20]. Determination of the GlnR regulon, by combining expression profiling of *M. smegmatis* wild type and a ∆*glnR* deletion mutant under nitrogen-starvation conditions in batch culture revealed a total of 103 genes directly controlled by GlnR [20]. A large portion of these genes (72 %) were also differentially expressed under nitrogen limitation in our continuous culture model, however, GlnR itself was not among them, indicating a posttranslational regulatory mechanism for GlnR like in *S. coelicolor* [13]. Comparison of the differentially expressed transcriptional regulatory proteins in batch culture versus continuous culture showed an overlap of six genes with four of these under control of GlnR (Table 3). We further observed 16 transcriptional regulatory proteins that were differentially expressed in continuous culture (Table 3). The TetR-like transcriptional regulator AmtR showed a 1.7-fold upregulation under nitrogen replete conditions and was therefore outside the selected cut-off. However, in our RNA-seq analysis we identified 127-fold downregulation of an antisense transcript (asRNA) of almost the entire *msmeg_4300* gene under nitrogen-depleted conditions (Additional file 6, Figure S3). These data suggest a mechanism of post-transcriptional regulation by an asRNA where transcription of *msmeg_4300* is enhanced upon nitrogen excess and modulates the translation efficiency of the AmtR encoding gene.

We identified several other cases of potential non-coding small RNAs that might regulate expression of genes involved in fatty acid and central carbon metabolism under nitrogen limitation (Additional file 7, Table S4). In our RNA-seq analysis, we observed a 102-fold downregulation of an asRNA of *msmeg_4299* gene under nitrogen depletion, while *msmeg_4299* showed a 2.7-fold upregulation under nitrogen limitation (Additional file 6, Figure S3). The intergenic region *msmeg_3131*-*msmeg_3132* comprises the promotor regions of the genes *msmeg_3131* (long-chain acyl-CoA synthetase) and *msmeg_3132* (DNA-binding protein) and revealed a 4.7-fold increase in transcription under nitrogen excess (Additional file 6, Figure S3). Interactions between sRNAs and the 5’UTR of mRNAs affect translation efficiency, while an interaction with the 3’UTR of mRNAs usually do not affect translation, but mRNA stability [36]. These findings are supported by a strong upregulation of genes involved in the degradation of fatty acids as well as the downregulation of genes involved in the TCA cycle as both metabolic pathways are CoA-dependent (Additional file 2, Table S1).

Interestingly, three regulatory proteins (one putative two-component system and two putative orphan response regulators) showed a different expression profile in continuous culture compared to batch culture. The operon *msmeg_6236*-*msmeg_6238* encodes for a two-component system with unknown function and the gene *msmeg_3297* encodes for a CadC-like transcriptional regulator that has been linked to pH homeostasis in *E. coli* [37]. No change in external pH was observed in our experiments, suggesting the CadC-like protein in *M. smegmatis* was performing a different function.

## Conclusions

**Fig. 4:**
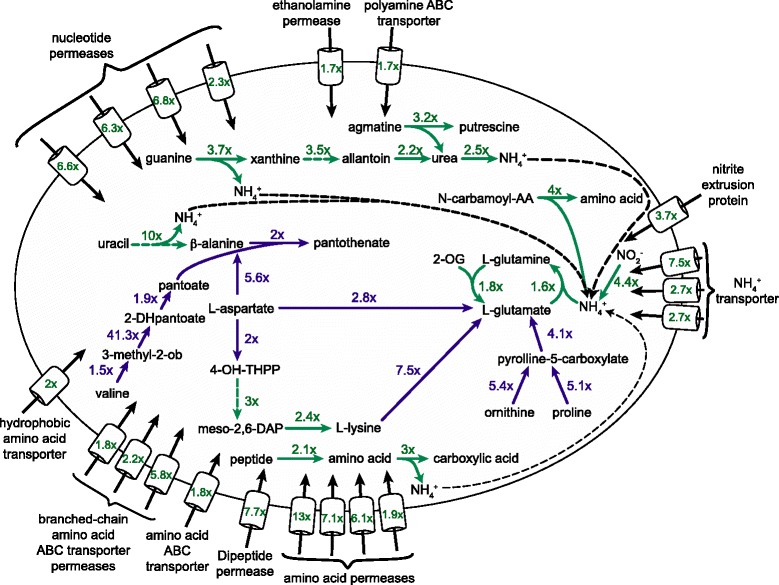
Overview of affected nitrogen uptake systems and metabolic pathways during nitrogen limitation in *M. smegmatis*. Shown are selected upregulated (cyan) and downregulated (purple) genes that are directly involved in nitrogen uptake, metabolism and ammonium assimilation. Fold change in gene expression and predicted directionality of reaction are indicated by numbers and arrows. 2-DHpantoate: 2-dehydropantoate; 2-OG: 2-oxoglutarate; 3-methyl-ob: 3-methyl-2-oxo-butanoate; 4-OH-THPP: 4-hydroxy-2,3,4,5-tetrahydrodipicolinate; AA: amino acid; meso-2,6-DAP: meso-2,6-diaminopimelate; NH_4_^+^: ammonium; NO_2_^−^: nitrite

Herein we report the transcriptomic response of *M. smegmatis* to nitrogen limitation in a continuous culture model at a defined growth rate (Fig. 4). We show that amino acid metabolism plays an important role in the adaptation of *M. smegmatis* to nitrogen depletion and identified 16 novel transcriptional regulators that were either directly or indirectly involved in the global transcriptomic response of *M. smegmatis* to nitrogen limitation. Several non-coding RNAs were differentially expressed suggesting transcriptional or post-transcriptional regulation of gene expression and we propose a regulatory mechanism involving a trans-acting asRNA for the AmtR protein in *M. smegmatis*. Comparison of our chemostat data to the global transcriptomic response in batch culture nitrogen run out experiments revealed that only 17 % of the previously described nitrogen-regulated genes overlapped between batch and continuous culture. Our data highlight the versatile metabolic capability of *M. smegmatis* and provide a molecular framework for understanding how environmental mycobacteria respond to nitrogen-depleted environments.

## Methods

### Bacterial strains, media and growth conditions

*M. smegmatis* mc^2^155 was grown in LBT or adapted HdB minimal medium supplemented with 0.2 % (w/v) glycerol (unless otherwise stated) as sole carbon source, NH_4_Cl in various concentrations as sole nitrogen source, 11.74 mM K_2_SO_4_ as sulphur source and 0.05 % (w/v) Tyloxapol at 37 °C with agitation (200 rpm). Aerobic starter cultures were inoculated to an initial optical density (OD_600_) of 0.05 and grown with agitation (200 rpm) at 37 °C. Chemostat bioreactors were prepared as previously described [23]. Samples to measure residual glycerol concentration and ammonium concentration were taken in intervals of four to six hours. Glycerol concentration was measured according to Garland and Randle [38] and ammonium concentration was measured according to Weatherburn [39]. After entering steady-state, the chemostat was left running for at least three volume changes before cell harvest.

### RNA extraction and Reverse transcriptase PCR

Cells were lysed by four cycles at 4800 rpm for 30s in a mini-Beadbeater (Biospec Products), with 30s on ice between each of the cycles. Total RNA was extracted using TRIzol® reagent (Ambion) according to the manufacturers instructions. DNA was removed by treatment with 3 U RNase-free DNase using the TURBO DNA-free kit (Ambion) according to the manufacturers instructions. The quality of the RNA was checked with the Bioanalyzer (RIN >9) and the concentration was determined using a NanoDrop ND-100 spectrophotometer.

Next, depletion of ribosomal RNA and strand-specific library preparation were performed, using the EpiCentre ScriptSEq™ Complete Kit for Bacteria according to manufacturers instructions. Quantification of nucleic acids was performed using a Qubit® Fluorometer to ensure DNA contamination of the samples was less than 10 % and libraries were created. Prepared libraries have undergone a quality control using an Agilent 2100 Bioanalyzer: DNA 1000 Labchip, Quant-iT dSDNA HS Assay for quantification and Quant-iT RNA Assay and Quant-iT Protein Assay for percentage contamination check, using an Invitrogen Qubit® Fluorometer. Libraries were run on a Illumina MiSeq 300 cycle Kit_v2 with a paired-end (PE) read length of 2x150 and a PhiX control library was also loaded and used as control for the run.

### Analysis of RNA sequencing data

Adapter sequences were removed from raw fastq files using Flexbar [40] and reads shorter than 50 bp were discarded. Sequences were then mapped against the *M. smegmatis* genome (GenBank NC_008596.1) using Bowtie2 with the”very sensitive” option. Counts for each gene, intergenic regions and counts on reverse strand were calculated with featureCounts [41], taking into account the reads’ strand-specificity. Statistical analysis and principal component analysis were performed using DESeq (Additional file 8, Figure S4 and Additional file 9, Figure S5) [42]. We considered differentially expressed genes based on their fold-change values as well as *p* values adjusted for multiple testing with the Benjamini-Hochberg procedure, which controls false discovery rate (FDR, referred to as *p*adj in DESeq results). Venn diagrams were generated using the BioVenn program [43]. Gene functions were assigned using public databases (NCBI [44], UniProt [45], KEGG [46], Biocyc Database Collection [47]).

### Supporting data

All RNA-sequencing data have been deposited in ArrayExpress and can be accessed through the accession number E-MTAB-3918. All other supporting data are included as additional files.

## Additional files

Additional file 1: Figure S1. Growth of *M. smegmatis* in batch culture with different carbon sources under nitrogen-depleted and nitrogen-replete conditions. (PDF 264 kb) Additional file 2: Table S1. A complete list of genes that are differentially expressed in response to nitrogen limitation in *M. smegmatis*. (XLSX 2413 kb) Additional file 3: Figure S2. Functional characterization of differentially expressed genes. (PDF 272 kb) Additional file 4: Table S2. Comparison of the gene expression profile between nitrogen-depleted continuous culture versus batch culture of *M. smegmatis*. (XLSX 88 kb) Additional file 5: Table S3. A list of genes that are differentially expressed in continuous culture, but not in batch culture. (XLSX 89 kb) Additional file 6: Figure S3. Images of IGV files from differentially expressed intergenic regions and antisense-strand reads mentioned in the text. (PPTX 495 kb) Additional file 7: Table S4. A list of differentially expressed intergenic and antisense-strand regions. (XLSX 12 kb) Additional file 8: Figure S4. Data quality assessment by sample clustering and visualization. (PPTX 147 kb) Additional file 9: Figure S5. Principal component plot of the samples as calculated from the variance stabilising transformation of the count data using DESeq and heatmap showing the Euclidean distances between the samples from the variance stabilising transformation of the count data using DESeq. (PPTX 72 kb)
