# Supplementary figures and images for: Defining the nitrogen regulated transcriptome of Mycobacterium smegmatis using continuous culture
Source: BMC Genomics. 2015 Oct 19;16:821. doi: 10.1186/s12864-015-2051-x (PMC4617892; doi:10.1186/s12864-015-2051-x)

[A]

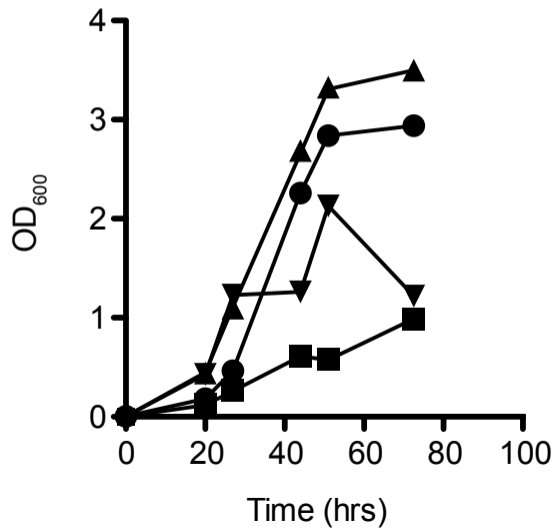

[B]

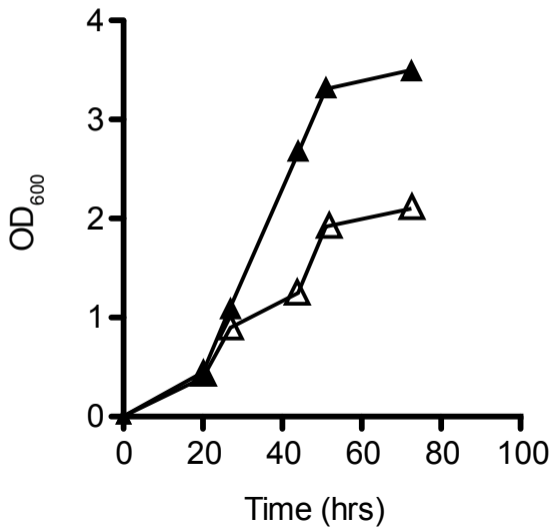

Supplement: Additional file 1: Figure S1. — Growth of M. smegmatis in batch culture with different carbon sources under nitrogen-depleted and nitrogen-replete conditions. (PDF 264 kb) [file 12864_2015_2051_MOESM1_ESM.pdf]

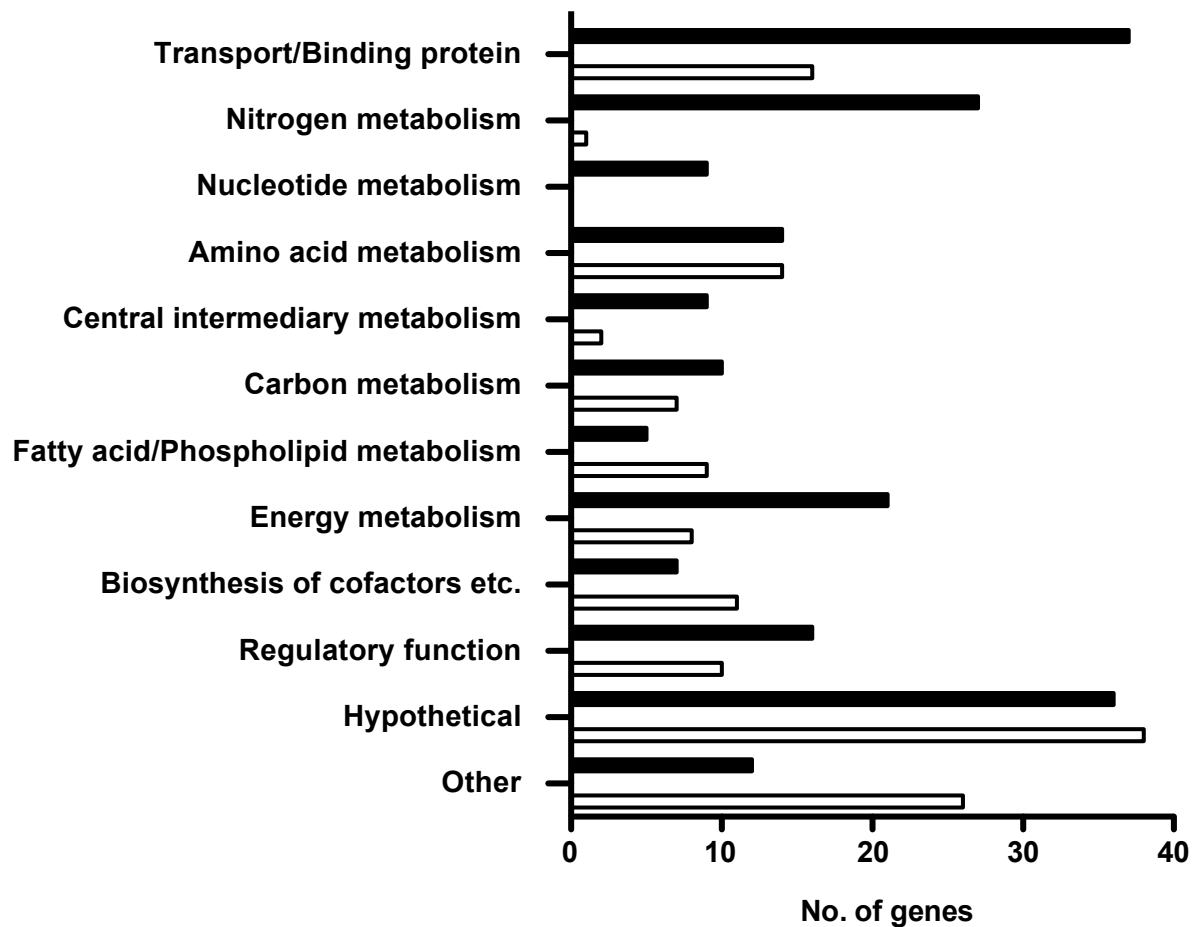

Supplement: Additional file 3: Figure S2. — Functional characterization of differentially expressed genes. (PDF 272 kb) [file 12864_2015_2051_MOESM3_ESM.pdf]

## Slide 1
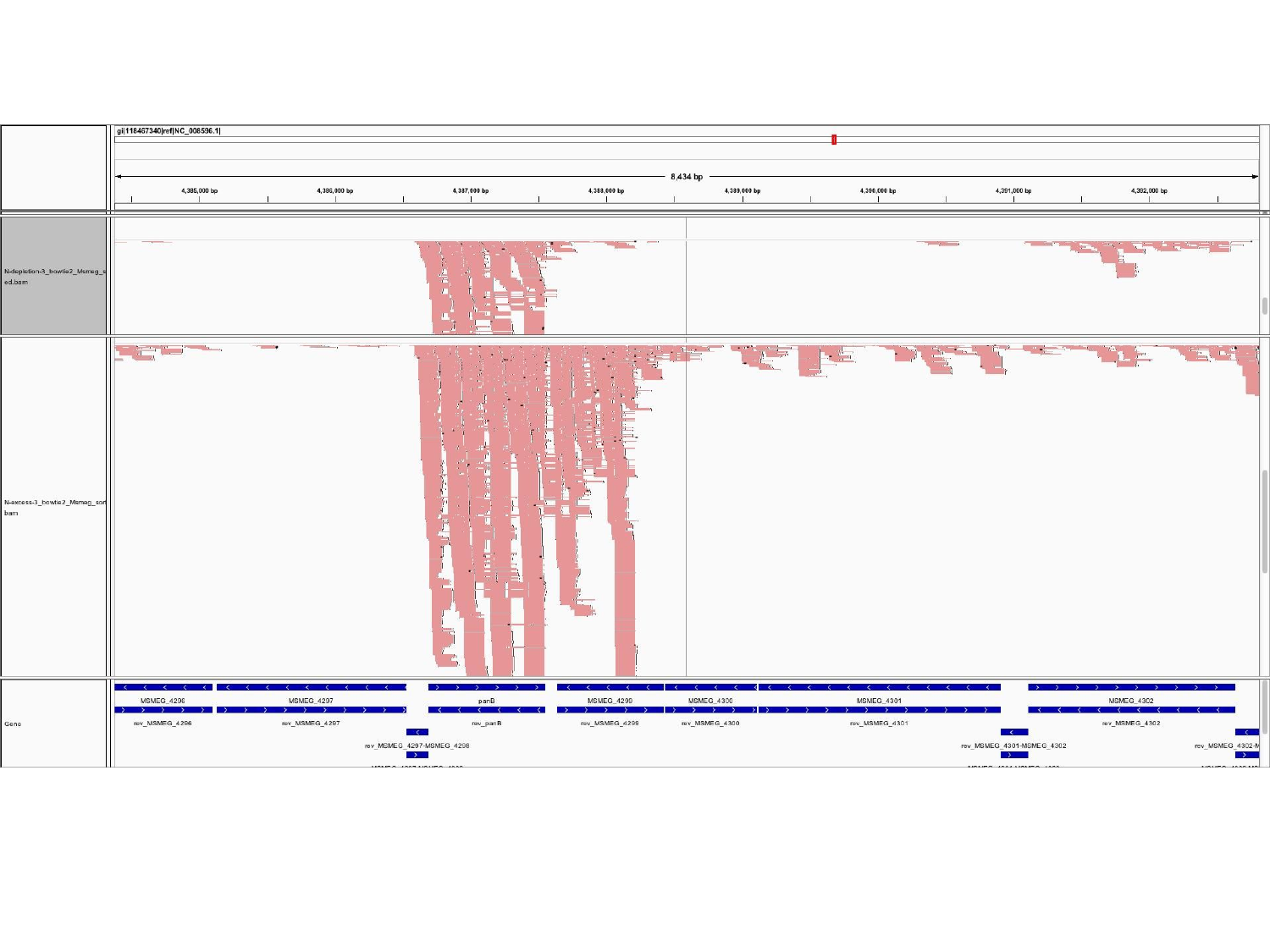

## Slide 2
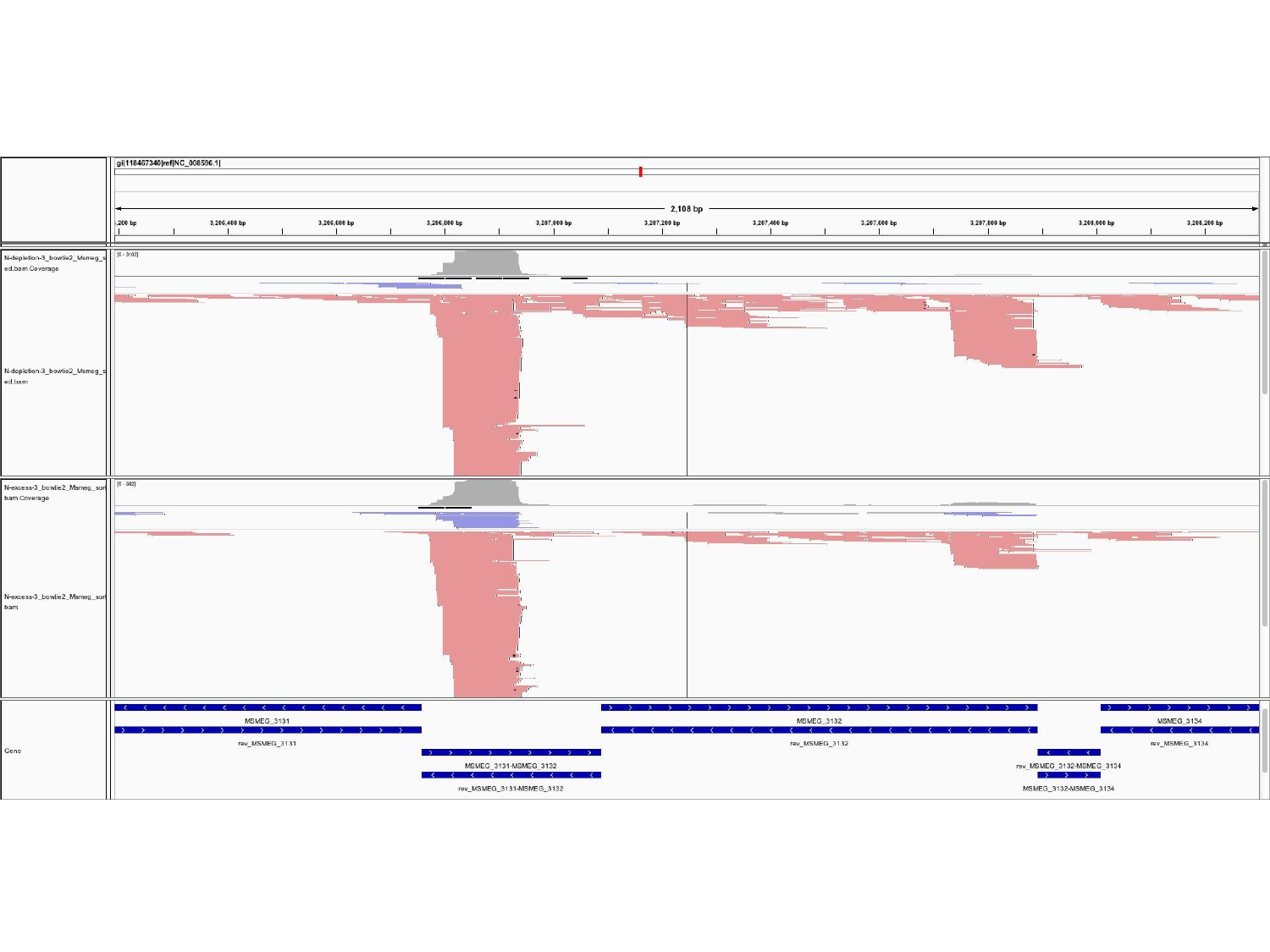

## Slide 3
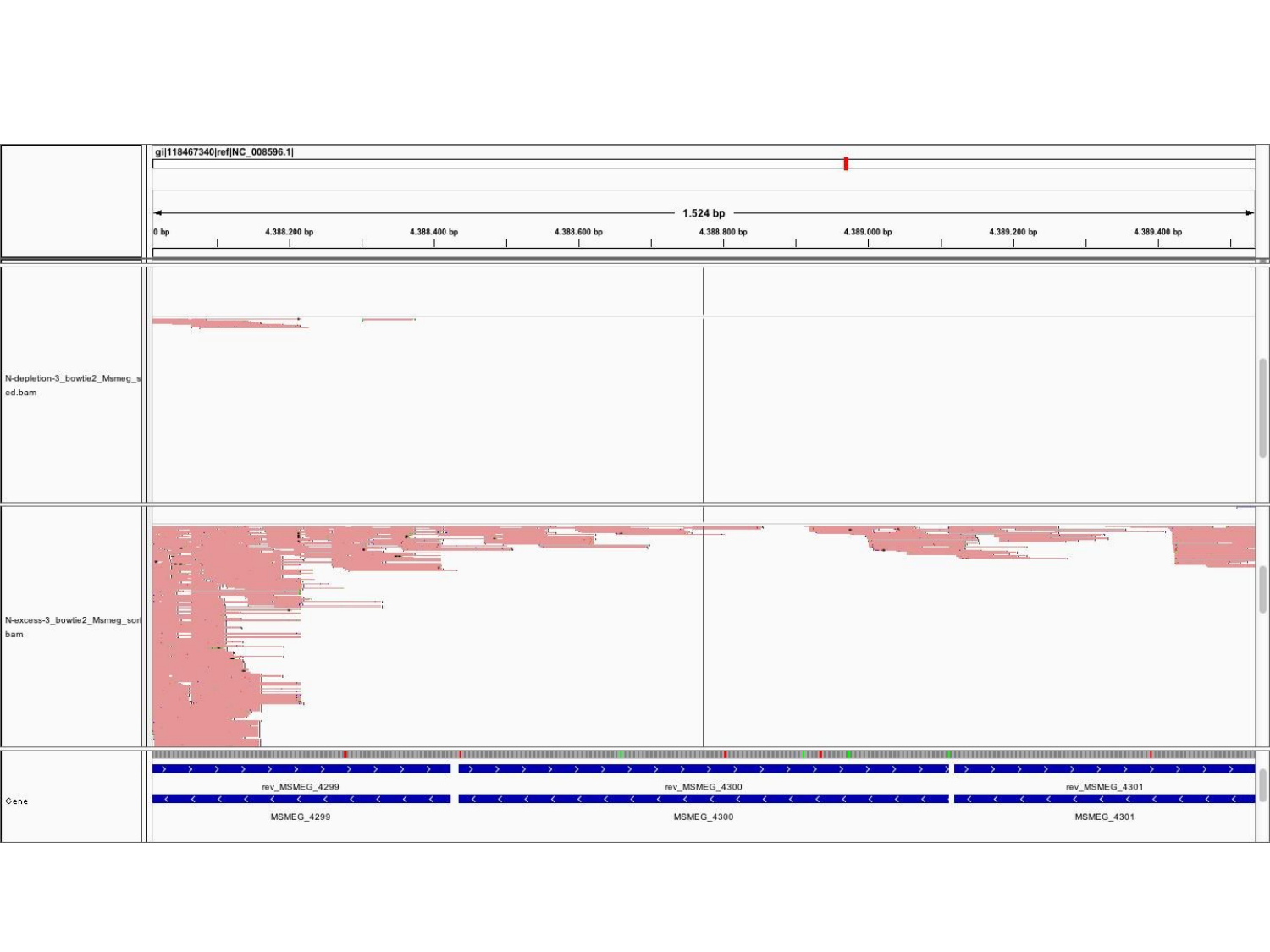

Supplement: Additional file 6: Figure S3. — Images of IGV files from differentially expressed intergenic regions and antisense-strand reads mentioned in the text. (PPTX 495 kb) [file 12864_2015_2051_MOESM6_ESM.pptx]
