# Supplementary material for: Defining the nitrogen regulated transcriptome of Mycobacterium smegmatis using continuous culture
Source: BMC Genomics. 2015 Oct 19;16:821. doi: 10.1186/s12864-015-2051-x (PMC4617892; doi:10.1186/s12864-015-2051-x)
Supplement: Additional file 8: Figure S4. — Data quality assessment by sample clustering and visualization. (PPTX 147 kb) [file 12864_2015_2051_MOESM8_ESM.pptx]

## Slide 1
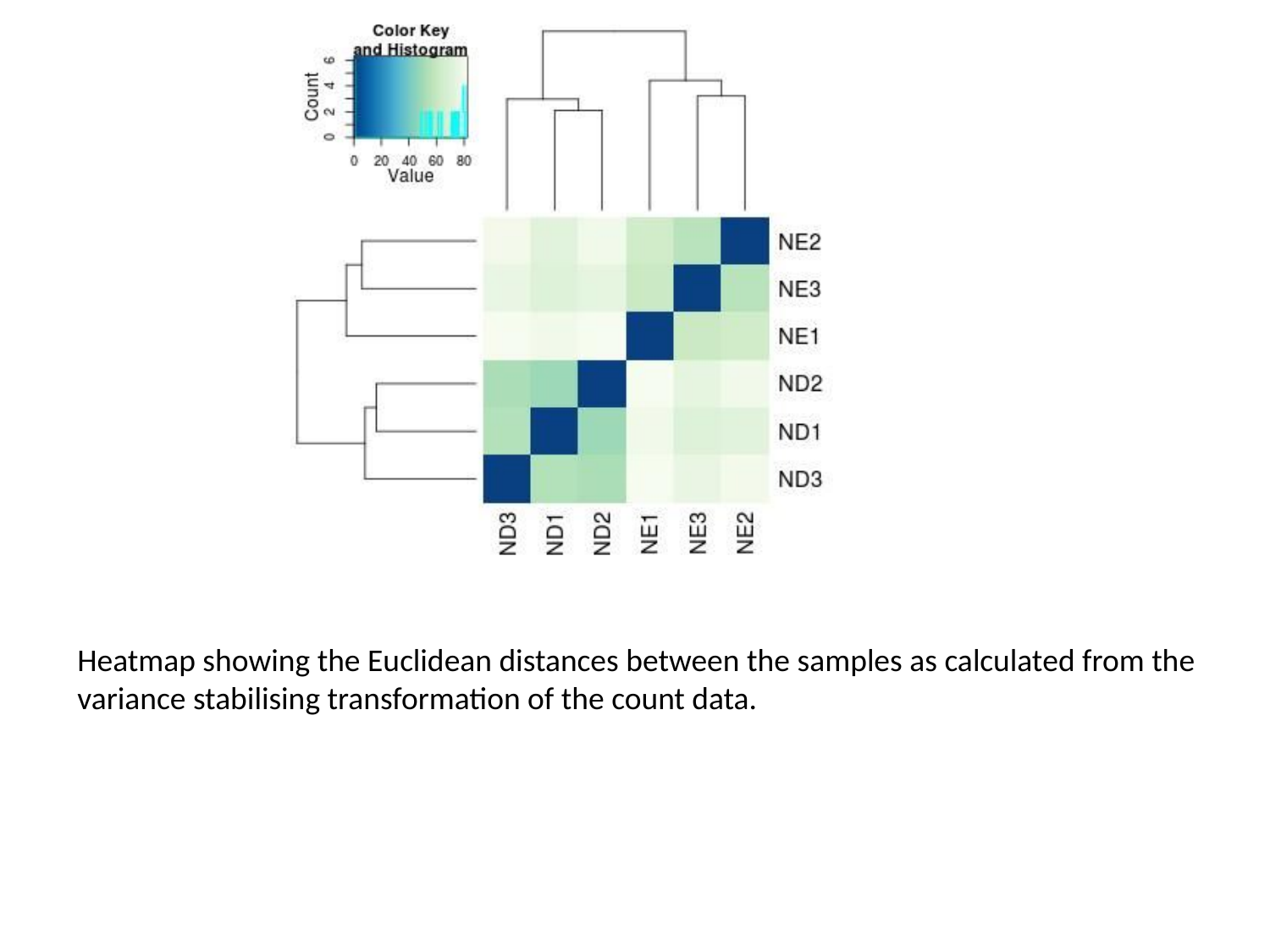

Heatmap showing the Euclidean distances between the samples as calculated from the variance stabilising transformation of the count data.

## Slide 2
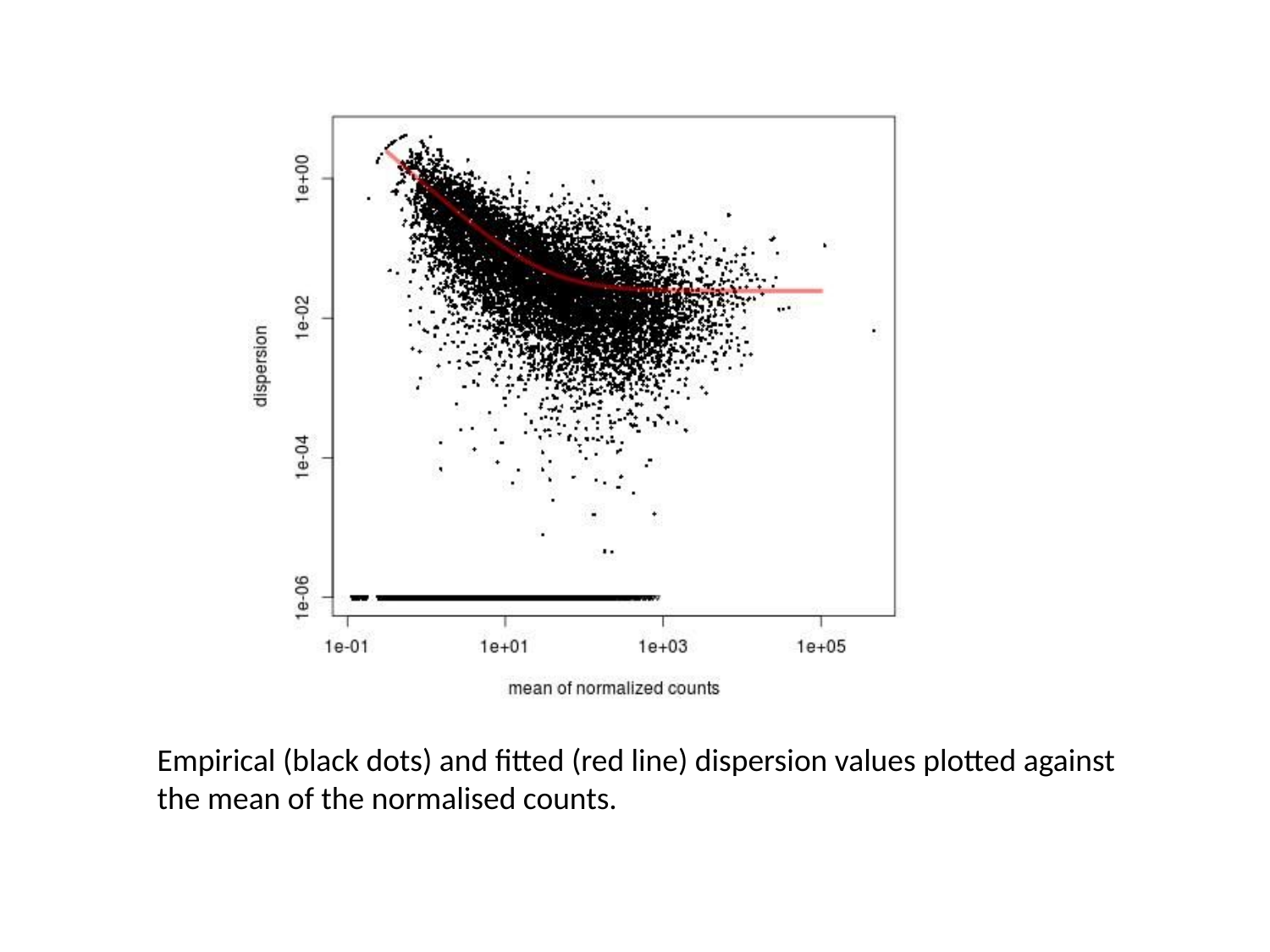

Empirical (black dots) and fitted (red line) dispersion values plotted against the mean of the normalised counts.

## Slide 3
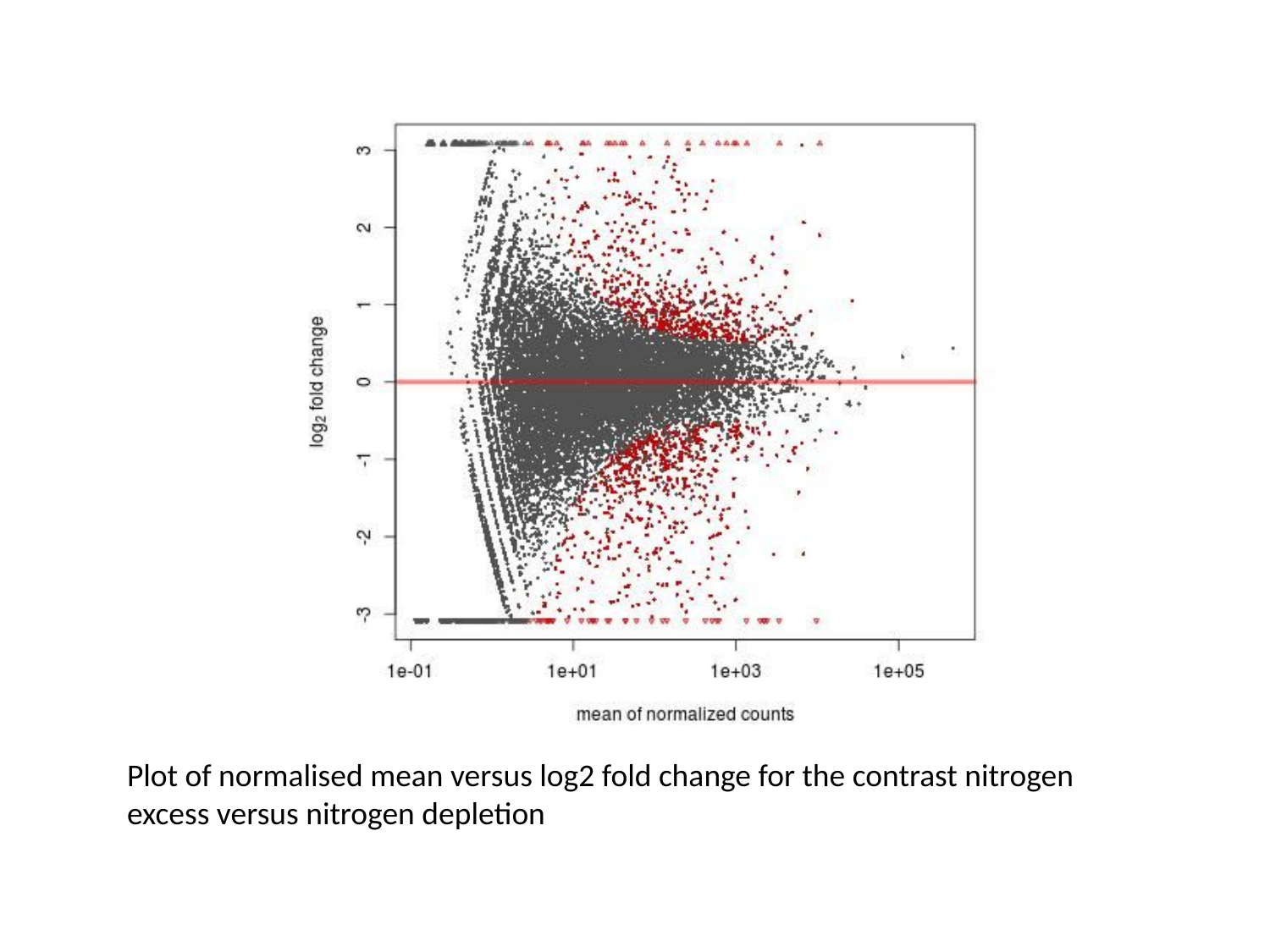

Plot of normalised mean versus log2 fold change for the contrast nitrogen excess versus nitrogen depletion
