# Supplementary material for: Defining the nitrogen regulated transcriptome of Mycobacterium smegmatis using continuous culture
Source: BMC Genomics. 2015 Oct 19;16:821. doi: 10.1186/s12864-015-2051-x (PMC4617892; doi:10.1186/s12864-015-2051-x)
Supplement: Additional file 9: Figure S5. — Principal component plot of the samples as calculated from the variance stabilising transformation of the count data using DESeq and heatmap showing the Euclidean distances between the samples from the variance stabilising transformation of the count data using DESeq. (PPTX 72 kb) [file 12864_2015_2051_MOESM9_ESM.pptx]

## Slide 1
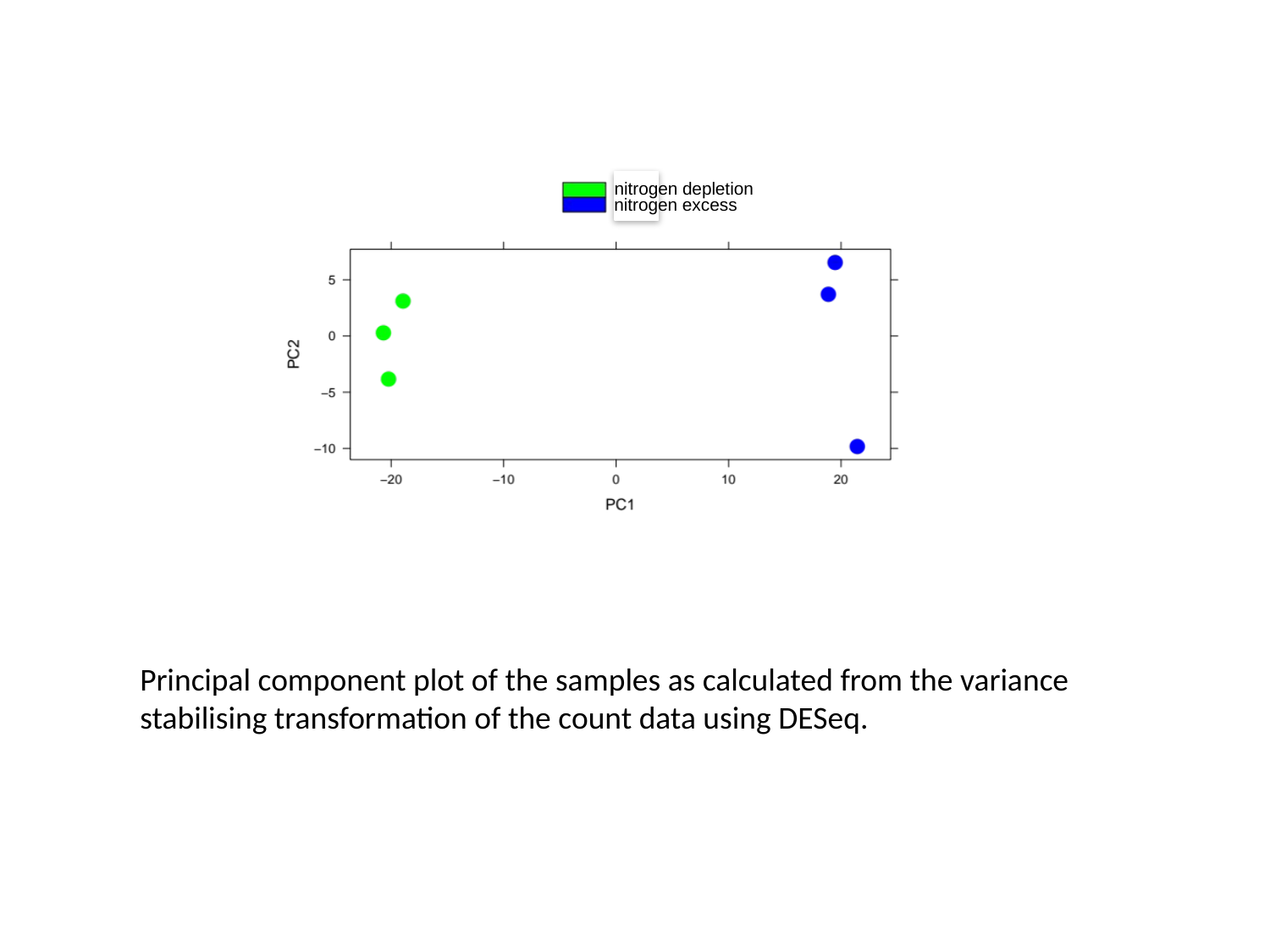

nitrogen depletion
nitrogen excess
Principal component plot of the samples as calculated from the variance stabilising transformation of the count data using DESeq.

## Slide 2
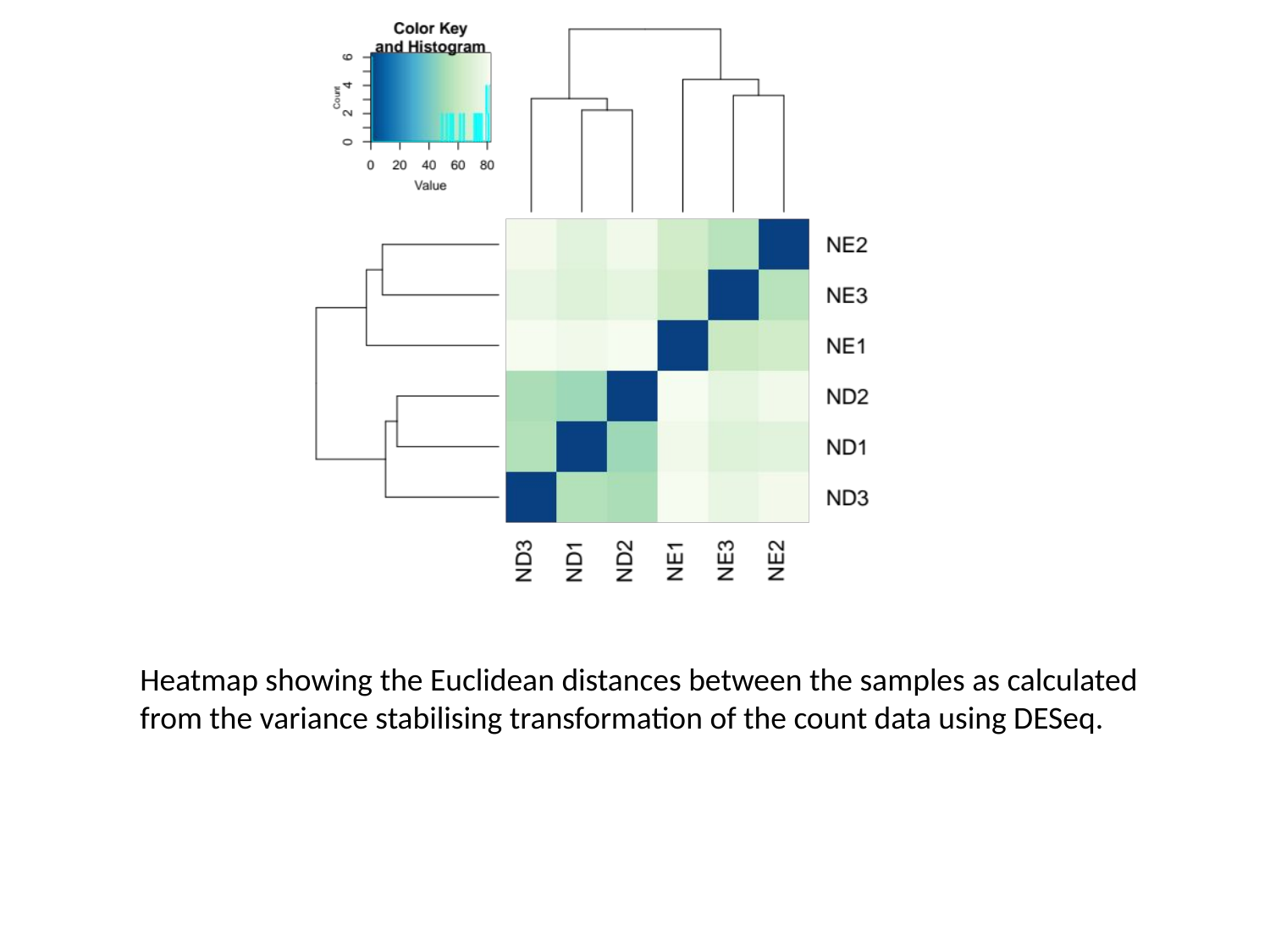

Heatmap showing the Euclidean distances between the samples as calculated from the variance stabilising transformation of the count data using DESeq.
